# RNF11 at the Crossroads of Protein Ubiquitination

**DOI:** 10.3390/biom10111538

**Published:** 2020-11-11

**Authors:** Anna Mattioni, Luisa Castagnoli, Elena Santonico

**Affiliations:** Department of Biology, University of Rome Tor Vergata, Via della ricerca scientifica, 00133 Rome, Italy; Anna.Mattioni@gmail.com (A.M.); Castagnoli@uniroma2.it (L.C.)

**Keywords:** Ring Finger Protein 11, HECT ligases, ubiquitination

## Abstract

RNF11 (Ring Finger Protein 11) is a 154 amino-acid long protein that contains a RING-H2 domain, whose sequence has remained substantially unchanged throughout vertebrate evolution. RNF11 has drawn attention as a modulator of protein degradation by HECT E3 ligases. Indeed, the large number of substrates that are regulated by HECT ligases, such as ITCH, SMURF1/2, WWP1/2, and NEDD4, and their role in turning off the signaling by ubiquitin-mediated degradation, candidates RNF11 as the master regulator of a plethora of signaling pathways. Starting from the analysis of the primary sequence motifs and from the list of RNF11 protein partners, we summarize the evidence implicating RNF11 as an important player in modulating ubiquitin-regulated processes that are involved in transforming growth factor beta (TGF-β), nuclear factor-κB (NF-κB), and Epidermal Growth Factor (EGF) signaling pathways. This connection appears to be particularly significant, since RNF11 is overexpressed in several tumors, even though its role as tumor growth inhibitor or promoter is still controversial. The review highlights the different facets and peculiarities of this unconventional small RING-E3 ligase and its implication in tumorigenesis, invasion, neuroinflammation, and cancer metastasis.

## 1. Introduction

The covalent attachment of ubiquitin to a variety of cellular proteins is a common post-translational modification (PTM) in eukaryotic cells. In addition to providing a signal for proteasomal degradation, ubiquitination is associated with different outcomes, from changing protein localization to modulating the biochemical properties of the target protein [1]. The conjugation process leads to the formation of an isopeptide bond between the C-terminus of ubiquitin and ε-amino group of a lysine residue [2]. Ubiquitin conjugation involves the sequential transfer of ubiquitin through an enzymatic cascade that consists of a ubiquitin-activating enzyme (E1), a ubiquitin-conjugating enzyme (E2), and a ubiquitin ligase (E3). E3 largely determines the high degree of specificity and selectivity for the target proteins and shows the greatest diversity among the ubiquitination machinery. RING (really interesting new gene) ligases, which represent the largest family of E3-ligases that consist of over 600 genes in human, are commonly considered as both scaffolds and allosteric activators of the conjugating E2 enzymes. Indeed, their RING domain brings in close proximity the specific E2 to the substrate and then promotes the direct transfer of ubiquitin from the E2 to the target protein [3]. Two other groups, called HECT (Homologues to the E6AP carboxyl terminus) and RBR (RING between RING) ligases, make up roughly 5% of the total number of known E3-ligases. The conjugation of ubiquitin by HECT and RBR E3-ligases takes place in two steps: ubiquitin is first transferred from the E2 to an active-site cysteine residue on the catalytic domain of the ligase and then the target substrate [4,5,6]. Despite their limited number, their well-known involvement in several human disease-related processes has called for a deeper understanding of their working mechanisms. 

Presently, different ubiquitination patterns have been characterized. A target protein can be modified by a single ubiquitin moiety on one or multiple sites (mono- and multi-ubiquitination) and/or by poly-ubiquitin chains, in which any of the seven Lys residues in ubiquitin (i.e., Lys6, Lys11, Lys27, Lys29, Lys33, Lys48, or Lys63), as well as the N-terminal amino group, can link the ubiquitin moieties in a chain [4,7,8]. The specification of the ubiquitin chain linkage lies both in E2 and in E3. In particular, some E2s are dedicated to specific ubiquitin linkages, while others are intrinsically more promiscuous. RING E3-ligases are intermediaries of the ubiquitination process; therefore, the specific isopeptide linkage within the poly-ubiquitin chain is established by the intrinsic features of the E2 enzyme participating in the reaction. As one E3 ligase is often able to bind different E2s, the same protein can be ubiquitinated by different E2/E3 combinations, which lead to different ubiquitination patterns [9,10]. On the contrary, the autonomy of the HECT ligases in the ubiquitination reaction makes them solely responsible for the determination of chain topology. Consistently, HECT ligases show a preference towards one or few ubiquitin chains. This preference is independent of the linkage specificity of the E2 enzyme acting in that ubiquitination cascade [9,10]. 

Each ubiquitination profile adopts a distinct structure that is specifically recognized by ubiquitin binding domains (UBDs) that are embedded in the ubiquitin effectors, which couple substrate modification to a downstream biological function. The capability of different ubiquitin effectors to determine specific functional outcomes explains the wide versatility of ubiquitination in different cellular pathways.

Finally, deubiquitinating enzymes (DUBs) process ubiquitin precursors, edit chain topology, or cleave ubiquitin off substrates, ensuring reversibility of the ubiquitination system [11]. The combination of these characteristics makes the ubiquitin system a powerful signaling network in the cell, that is highly regulated at multiple levels in order to exert its function in a spatially and temporally controlled fashion.

## 2. RNF11, A Multifunctional RING-H2 Ligase 

### 2.1. Domain Organization and Cellular Localization of RNF11

**Figure 1:**
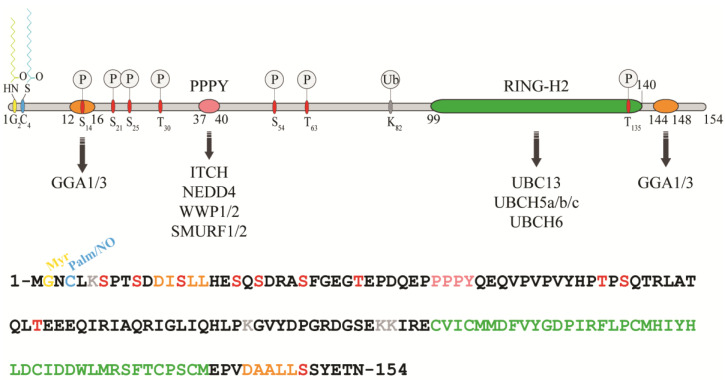
Protein sequence, domain organization and binding partners of the RING-H2 E3-ligase RNF11. Residues identified as target sites for post-translational modifications are shown. Colours legend: the PPY motif is coloured in pink, the RING-H2 domain in green, the myristoylation site is in yellow, the palmitoylation/nitrosylation site in light blue, the di-leucine motifs are in orange, the phosphorylation sites are in red, and the lysine residues are in grey.

RNF11 is a 154-amino acids protein that contains a canonical RING-H2 finger motif (C3H2C2, 99–140aa) at the carboxyl-terminal end (Figure 1). The RING-H2 domain appears to be a stable folded monomeric domain showing structural similarities with the RING1 domain of RBR E3s [12]. The full-length protein contains disordered regions, which mainly involving the N-terminal end and the short C-terminal tail following the RING domain (aa 141–154) [12]. However, the structure of both terminal ends is predicted to be highly influenced by the presence of post-translational modifications and the interaction with binding partners (see below).

The remarkable aspect of RNF11 is the presence of a PY (37-PPPY-40) motif upstream to the RING-H2 domain that allows for RNF11 to interact with the WW-domains containing proteins of the NEDD4 family of HECT-E3 ligases (among them: ITCH, NEDD4, SMURF1/2, and WWP1/2) [13,14,15,16,17,18]. This feature makes RNF11 the only RING ligase potentially capable of interacting with all the members of the NEDD4-like family. In addition, a putative ubiquitin-interacting motif (UIM) (corresponding to residues 67–74), located between the PY and the RING-H2 motif, has been predicted [19]. Nevertheless, the presence of a glycine (Gly) instead of the highly conserved serine (Ser) makes the effective functionality of this motif controversial. Accordingly, recent data show that the UIM of RNF11 does not bind ubiquitin in vitro, which leaves open the question whether it acts as a functional ubiquitin binding site in vivo [12]. Because the non-covalent interaction between E3 enzymes and ubiquitin has clearly been shown to influence the enzymatic activity of almost all the main classes of ligases [20,21,22,23,24,25,26], clearly defining the ability of RNF11 to bind ubiquitin would provide useful hints regarding its regulatory mechanism. 

In growing cells, RNF11 shows an intracellular vesicular distribution, which, in a steady state, mainly corresponds to the early and recycling endosomes. The access to these compartments appears to be strictly regulated by multiple sorting determinants. The N-terminal end of RNF11 undergoes a double acylation during protein maturation [17]. Immediately after the removal of the first methionine, which takes place during translation, a myristoyl group is covalently and irreversibly attached to Gly2 by an N-myristoyl transferase, followed by palmitoylation of the proximal cysteine 4 (Cys4), a reversible modification that occurs in the Golgi compartment [27,28]. The double N-terminal acylation is a pre-requisite for membrane localization [17]. Additional signals and protein-protein interactions allow for the intracellular trafficking of RNF11 and its access to the early and recycling endosomes [17]. More specifically, two acidic di-leucine motifs are the sorting determinants that drive the intracellular traffic of RNF11. The N-terminal motif (15-LL-16) is preceded by two aspartic residues, Asp11 and Asp12. These residues give rise to two partially overlapping acidic di-leucine sorting motifs: the DxxxLL consensus sequence (residues 11–16) mediates the interaction with the clathrin adaptor protein 2 (AP-2) complex, while the DxxLL motif (residues 12–16) interacts with the VHS (Vps27p, Hrs, and STAM) domains of the Golgi-localized, gamma adaptin ear-containing, ARF-binding (GGA) protein family. The di-leucine couple acts as a sorting signal at both the trans-Golgi Network (TGN) and at the plasma membrane. Conversely, a C-terminal acidic di-leucine motif (144-DAALL-148) seems to rather regulate the distribution of RNF11 inside the endosome compartment, between the early endosome and the recycling routes [18]. 

Data from two-hybrid screenings and biochemical validations have clearly shown that, in addition to GGA proteins, RNF11 directly interacts with resident proteins of the early and recycling endosome compartments, such as HRS (Hepatocyte growth factor-regulated tyrosine kinase substrate), STAM2 (Signal Transducing Adaptor Molecule 2), EPS15 (Epidermal Growth Factor Receptor Pathway Substrate 15), SARA (SMAD anchor for receptor activation), and ANKRD13A [29,30]. Nevertheless, none of these proteins appear to be uniquely responsible for RNF11 localization. Therefore, it is far more likely that RNF11 cycling in endosomal compartments results from the interaction with different binding partners, in a redundant mechanism that increases system resilience.

Interestingly, the partial and transient re-localization of RNF11 has been described upon cell stimulation by the EGF ligand [31]. Soon after induction, RNF11 positive signals move toward the cell periphery and the plasma membrane, while prolonged stimulation results in the RNF11 translocation to the nucleus. IGF treatment also leads to nuclear RNF11 accumulation [32]. In both cases, this translocation requires PI3K/Akt signaling, but further investigation is needed in order to unravel the underlying mechanisms [32]. Evidence that some nuclear RNF11 accumulation has been observed in lung, renal, and head and neck cancers [33] highlights the importance of further examination.

### 2.2. RNF11 Post-Translational Modifications and Functional Consequences

In addition to acylation, several other post-translational modifications occur on RNF11, which affect both stability and biological functions (Table 1). 

#### 2.2.1. S-Nitrosylation

It has been found that nitric oxide (NO) can be reversibly attached to the thiol group of the cysteine 4 residue (Cys4) of RNF11, which determines an increase of in vitro RNF11 autoubiquitination activity [34]. Interestingly, S-nitrosylation and S-palmitoylation appear to be mutually exclusive, suggesting intriguing consequences on RNF11 biology. Indeed, cycles of palmitoylation and nitrosylation could allow for the interaction with a wider variety of binding partners in response to specific signals. Moreover, given that S-nitrosylation affects the localization of nuclear proteins that are involved in the transcriptional regulation [35], the recent observation that RNF11 also performs transcriptional functions [29,32] (see below) would suggest that the dynamic exchange between different post-translational modifications, occurring at the N-terminal end of RNF11, orchestrates shuttling between nucleus and cytoplasm. Further investigation is required in order to clarify the physiological impact of this modification on RNF11 biology.

#### 2.2.2. Ubiquitination

Four lysines (Lys6, Lys82, Lys94, and Lys95) are putative sites for RNF11 ubiquitination. Among them, Lys82 in murine Rnf11 has been found to be ubiquitinated in the liver, brain, and heart, but not in kidney and muscle [36], which suggests the existence of district-specific RNF11 ubiquitination patterns that may contribute to modulating RNF11 biological functions and stability. The degradation of RNF11 protein seems, in fact, to be primarily proteasome-dependent and ubiquitin-mediated. The functional inactivation of the RING domain severely reduces RNF11 protein levels, while the inactivation of the binding site for the HECT E3-ligases (Tyr40Ala) strongly promotes protein stability by abrogating RNF11 ubiquitination [17]. Even though these data clearly support a role for ubiquitination in regulating RNF11 stability, the underlying mechanism is still unclear. To date, functional interactions with the RING domain of RNF11 have been validated for the E2 enzymes UbcH5, UbcH6, and Ubc13 (also called UBE2N) [29,37]. However, while Malonis and colleagues reported the autoubiquitination activity of RNF11 [38], Chen and collaborators have not observed it in in vitro assays. Thus, the functionality of RNF11 RING domain and the mechanism by which it contributes to RNF11 stability both remain controversial [39].

On the other hand, several E3-ligases have been identified as interactors of RNF11 and have been reported to mediate its ubiquitination. Whether these enzymes exert a proteolytic role or mainly a regulatory function is still a subject of investigation. In fact, only the overexpression of NEDD4, which weakly promotes RNF11 ubiquitination, has been reported to be associated with a reduction of RNF11 protein levels [39]. Instead, the overexpression and knock-down of the E3-ligases ITCH, WWP1, and SMURF2 both strongly affect RNF11 ubiquitination, but not its protein levels. Therefore, these modifications do not seem to target RNF11 for degradation but may rather serve regulatory functions. Coherently, the observation that a Lysine-less version of RNF11 accumulates in the Golgi significantly more than the wild-type protein [38] further suggests that ubiquitination might tightly regulate, in space and time, RNF11 functions.

Notably, it has been reported that the mutant of RNF11 inactivated in the canonical binding site for HECT E3-ligases is still ubiquitinated by ITCH. This suggests the presence of additional non-canonical PY motifs in RNF11 that might account for different RNF11 ubiquitination patterns that are potentially linked to different functional outcomes. Many other E3-ligases have been identified as putative RNF11 binding partners [40]. Nevertheless, their impact on RNF11 ubiquitination and stability has not yet been investigated.

#### 2.2.3. Sumoylation

Recently, RNF11 has been found to be ubiquitinated and sumoylated in two human acute myeloid leukemia (AML) cell lines. Interestingly, RNF11 also showed highly significant differences in its sumoylation levels between parental and resistant cell lines, making it potentially one of the highest predictive biomarkers of AML chemoresistance [41]. Because dysregulation only affects specific enzymes and not the entire Ub/SUMO pathways, unravelling the physiological role of such modification on RNF11 biological properties is expected to have a significant impact on the comprehension of the mechanisms of chemo-resistance development.

Moreover, SUMO-dependent processes can regulate the nuclear transport of proteins [42]. Therefore, as hypothesized for S-nitrosylation, sumoylation could regulate the nuclear import of RNF11 in specific cellular conditions. Future investigations will clarify the impact of this post-translational modification on the regulatory mechanisms that govern the nuclear functions of RNF11.

#### 2.2.4. Phosphorylation

RNF11 appears phosphorylated when stably or transiently expressed in cells [18]. The residue/s involved has/have not been clearly determined, but several serine (Ser, S) and threonine (Thr, T) residues have been indicated as potential phosphorylable sites by different protein kinases. To date, only the phosphorylation of Thr135 (T135) by the Protein Kinase B (PKB)/AKT has been validated both in vivo and in vitro [29]. This phosphorylation generates a functional binding site for 14-3-3 proteins and it has been associated with the nuclear translocation of RNF11. Accordingly, in the presence of constitutively active PKB, RNF11 accumulates in the nucleus [29].

Experimental data reporting the identity of other kinases that are involved in RNF11 modification are still missing. Nevertheless, further information about phosphorylation sites in RNF11 can be retrieved by mass spectrometry-based phosphoproteomic data collected in public curated databases (Phosphosite [19] and Phospho.ELM [43]) and by using server predictors (NetPhos 20.0 [44]) (Table 1). Twelve phosphorylated residues have been reported in mouse and/or human RNF11, showing different patterns in distinct tissues [45,46,47,48,49]. Most of them are clustered at the N-terminal end. In particular, the S14 lies within the di-leucine sorting signal that is essential for the correct intracellular localization of RNF11. This suggests that the RNF11 phosphorylation profile could modulate the temporal and spatial accessibility to its sorting motifs. Other six potentially phosphorylable Ser residues are clustered in the first 25 residues of RNF11, which are expected to be in close proximity with the plasma membrane. In this case too, the addition of negative charges could represent a key mechanism regulating RNF11 membrane anchoring and the dynamic of its intracellular distribution. For example, it has been shown that, following Protein Kinase C (PKC)-dependent phosphorylation, the K-ras protein rapidly dissociates from the plasma membrane and migrates to intracellular compartments [50]. Similarly, phosphorylation/dephosphorylation cycles, which are triggered by different cellular stimuli, might strictly regulate the subcellular localization of RNF11 subcellular and in turn its functions.

### 2.3. The RNF11 Interaction Network

An important clue to understand the biological role of RNF11 comes from data describing the RNF11 proteome, which were collected by both Yeast-two-Hybrid (Y2H) and mass spectrometry approaches (Table 2) [30,32,39]. By comparing the data that were collected from these approaches, we can extract useful information about RNF11 in terms of both molecular and biological functions.

First, RNF11 appears to be a common interactor of almost all members of the NEDD4 family. This is the best characterized family of HECT-E3 ligases and includes nine members in humans (NEDD4, NEDD4L, ITCH, WWP1, WWP2, SMURF1, SMURF2, NEDL1/HECW1, and NEDL2/HECW2) typified by the presence of C2 and WW domains located upstream of the catalytic HECT domain [51]. The C2 domain binds membrane phospholipids and it is involved in the recruitment of the HECT ligases to endosomal membranes [52]. The WW domains, which range from two to four copies, mediate the recognition of substrates by binding to proline sequences in the target protein [53,54]. As expected, most of the interactions between RNF11 and the NEDD4 family members result to be stably associated with intracellular membranes [38] and involve the WW domain/PPxY recognition, at least where the interacting region can be retrieved from the experimental approach. Most of the interactions with the NEDD4 family members have been experimentally validated, thus definitely linking the biological role of RNF11 to one of the most studied E3-ligase families [13,15,17,38].

In addition, the Y2H screening identified the ligases HUWE1, HERC1, and HERC2 as potential RNF11 interactors [29]. They belong to the so-called group of “other” HECT ligases, because, apart from the catalytic HECT domain, they do not have a unique and recognizable domain content [55]. The binding region selected in HUWE1 does not show domains or motifs already characterized, while both HERC1 and HERC2 interacted with RNF11 by means of a Ubiquitin Associated (UBA)-like domain. HUWE1 was also confirmed to be an RNF11 binding partner by SILAC-based AP-QMS approach [31]. Finally, two RING E3-ligases, LISTERIN and hRUL138, were isolated as potential membrane-associated RNF11 interacting proteins by mass-spectrometry [38].

Along with the E3-ligases, several E2 enzymes have been identified, in line with the presence of a RING-H2 domain in RNF11. The list includes UbcH5a, UbcH5b, UbcH5c, UbcH5d, UbcH6, UbcH7, UbcH9, HBUCE1, UBE2V1, and UBE2N. Some of these interactions have been confirmed in at least two screening approaches. The functional interaction of RNF11 with UbcH5, UbcH6, and UBE2N enzymes has also been validated via in vitro auto-ubiquitination assays [53].

A third group of RNF11 binding partners includes zinc-finger containing proteins (TNFAIP3, RABEX5, NDP52, TAX1BP1, OPTN, and NEMO). Interestingly, with the exception of Rabex5, in which a Zinc Finger (ZnF) domain is in close proximity to the selected binding region, the RNF11 binding regions include at least one ZnF motif. Finally, a fourth group shares the presence of a ubiquitin-binding motif in the region selected in the Y2H screening. A variety of UBD categories can be identified: UIM (EPN1-4, ANKRD13A, STAM2), GAT (GGA1, GGA3, TOM1L2), UBA (RP42, UBQL-4, UBQL-2 and USP5), CUE (FLJ1588 and AUP1), and MIU-MyUb (MYO6). Additionally, the UBD-containing protein ERCC6, the UBZ-containing protein (SPRTN) and the new CUBAN domain, which is only found in the KHNYN protein [54], have also been identified. Therefore, the data indicate that the large majority of preys identified in the Y2H screening have been selected by virtue of the presence of ubiquitin binding domains in their primary sequence. Conversely, only a limited number of interactors identified by mass-spectrometry contain ubiquitin-binding domains. The simplest way to explain so many different UBD-mediated interactions retrieved from Y2H screening would be to state that RNF11 is ubiquitinated by the endogenous yeast HECT ligase Rsp5, following a typical PPPY/WW domain recognition, therefore allowing for the recruitment of UBD-containing interactors selected by virtue of the covalent modification. On the other hand, when further studying the interaction between RNF11 and the ANKRD13 family of UIM-containing proteins, identified by both Y2H and SILAC approaches, we found that RNF11 directly contacts the UIM region of ANKRD13A protein and this recognition does not require RNF11 ubiquitination [31]. Accordingly, point mutations that inactivate the ubiquitin binding properties of the ANKRD13A UIM motifs abrogate the in vitro interaction with the RING protein. This intriguing observation leaves open the possibility that, as for ANKRD13A, other UBD-mediated interactions that are identified by Y2H could be based on a peculiar recognition pattern present on RNF11, which would “mimic” the UBD binding surface in the ubiquitin molecule.

The RNF11 protein–protein interaction network, obtained from these three screenings, has been analysed for the biological functions of the putative binding partner, confirming the involvement of RNF11 in cellular trafficking processes and in several signaling pathways.

### 2.4. RNF11 in Intracellular Traffic, Signal Transduction and Oncogenesis

Within higher eukaryotic cell membranes, traffic pathways connect various membrane organelles and maintain cellular homeostasis by retaining the correct complement of proteins and lipids. The exocytic/secretory pathway carries newly synthetized proteins and lipids from the endoplasmic reticulum (ER) through the Golgi to the plasma membrane. The endocytic pathway internalizes proteins from the extracellular environment or from the plasma membrane to the early endosomes. Here, cargos are either sorted to the recycling endosome, to be brought back to the plasma membrane, or they are delivered to the late endosomes, and finally to the lysosomes for degradation. Membrane-bound vesicles mediate these trafficking pathways, and sophisticated machineries, as well as complex mechanisms of regulation are required in order to ensure efficiency and specificity in the selection and delivery of cargos.

Several lines of evidence strongly support the notion that RNF11 does not act simply as a cargo of membrane adaptors, but it actively participates in controlling membrane traffic processes. Notably, RNF11 gene silencing results in a strong inhibition of ER-to-plasma membrane transfer of the well-characterized secretory cargo membrane protein tsO45G, without affecting the morphology of the ER and Golgi complex [56]. The molecular mechanism behind this evidence is still unknown; however, it has been recently reported that, under continuous Epidermal Growth Factor (EGF) stimulation, the overexpression of RNF11 up-regulates the mRNA levels of SEC23B, SEC24B, and SEC24D, which are components of the ER export machinery specifically required for the ER export of newly synthetized EGFR(Epidermal Growth Factor Receptor). On the contrary, RNF11 knockdown down-regulates them [32]. Understanding how RNF11 regulates the levels of these proteins needs further investigation. However, there might be a possible direct role of this E3-ligase in gene transcription. Indeed, RNF11 has been shown to bind DNA [15], and prolonged EGF treatment has resulted in the nuclear translocation of RNF11 [32].

Other data suggest a regulatory role for RNF11 in endocytic trafficking. The overexpression at middle and high levels of wild-type RNF11 causes the early endosome vesicles to swell. Moreover, cells overexpressing the RING domain inactive mutant of RNF11 lose the integrity of early and late endosomes and show a severe impairment of transferrin uptake [18]. Finally, Kostaras and colleagues demonstrated that RNF11 silencing decreased the degradation of activated EGFR and increased EGF recycling [30,57]. Similar effects have been described as a consequence of the silencing of well-known components of the trafficking machinery [57,58,59], which suggests that RNF11 could also be part of it.

The endocytic route is essential for the signal termination of membrane receptors and is strictly regulated by ubiquitination at different levels. Once activated by their cognate ligands, receptors are rapidly ubiquitinated. This represents a crucial signal, as it sorts receptors to lysosomes for degradation via the Endosomal Sorting Complex Required for Transport (ESCRT) pathway [60]. This pathway comprises four multi-subunit protein complexes (ESCRT-0, -I, -II, -III), which act in concert to direct ubiquitinated receptors along the degradative route [61]. It has been proposed that RNF11 participates structurally and functionally to the ESCRT-0 complexes. The E3-ligase interacts with the ESCRT-0 subunits HRS (Hepatocyte growth factor-regulated tyrosine kinase substrate), EPS15 (epidermal growth-factor receptor substrate protein 15), and STAM1/2 (signal transducing adaptor molecule 1/2) [14]. Moreover, RNF11 interacts with the SMAD anchor for receptor activation (SARA) protein, which possibly mimics the function of HRS by assembling an alternative ESCRT-0 complex [62]. In this context, RNF11 may have a role as a fine tuner of ubiquitination. Indeed, the sorting activity of ESCRT complexes is strictly controlled by the ubiquitination of their subunits. Many of these proteins are subject to mono- or poly-ubiquitination that is predicted to induce an autoinhibitory conformation preventing the ESCRT from recognizing ubiquitinated cargos. Thus, the regulation of the ubiquitination status of the sorting machinery is crucial for the efficient sorting of activated receptors to the lysosome and for the consequent signal termination.

Several HECT ligases, in concert with many deubiquitinating enzymes, have been shown to modulate the ubiquitination of proteins that belong to the sorting machinery. RNF11 interacts with several of these E3-ligases, in particular those of the NEDD4 family, counteracting their activity. In fact, we have shown that ITCH overexpression promotes the ubiquitination of the ESCRT-0 subunits HRS and STAM2, as well as of the endocytic adaptor proteins GGA1/3 and ANKRD13A [18,31]. However, the concomitant overexpression of RNF11 reverts this result, lowering the ubiquitination status of these endocytic adaptor proteins. This outcome is strictly dependent on the RING activity, as the overexpression of the RING inactive mutant exacerbates ITCH-mediated ubiquitination of these proteins [18].

A similar mechanism has been described for the RING-domain E3-ubiquitin ligase deltex-3-like (DTX3L). By inhibiting ITCH activity, DTX3L maintains the ESCRT-0 subunits HRS and STAM-1 moderately ubiquitinated, so that they are able to efficiently sort the ubiquitinated chemokine receptor CXCR4 to lysosomes for degradation and signal termination [63].

In the last years, the function of RNF11 has been potentially linked to a plethora of different cell stimuli. Even though we still are not able to link these experimental evidences to a clear molecular mechanism, some preliminary observation can help in paving the way.

#### 2.4.1. The TGF-Beta Signaling Pathway

The signaling pathway of the transforming growth factor beta (TGF-β) is one of the major regulators of cell communication in the adult organism as well as in the developing embryo. It is involved in the regulation of crucial cellular processes, such as cell growth, differentiation, apoptosis, and cellular motility. Alterations in TGF-β signaling have been implicated in many diseases, particularly cancer, where it plays a dual role as tumor suppressor in early-stage disease and tumor promoter in advanced malignant disorders [64].

**Figure 2:**
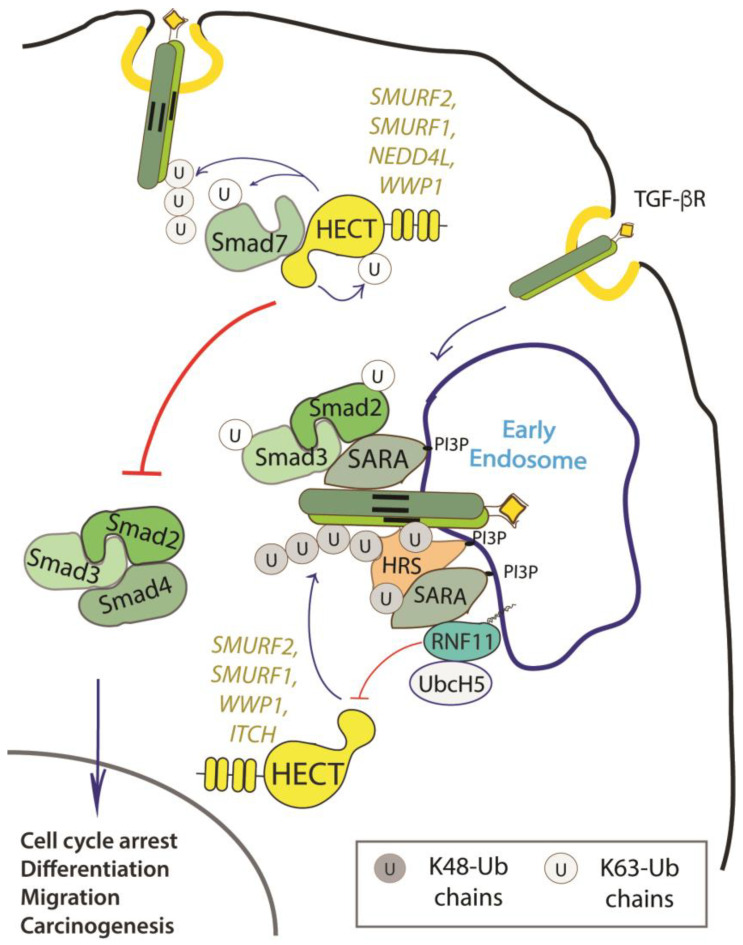
Role of RNF11 in the transforming growth factor beta (TGF-β) signaling pathway. Following ligand binding, the ubiquitination of the TGF-b receptor promoted by the SMURF2-SMAD7 E3 ligase complex targets the receptor to the lysosome or the proteasome for degradation. From the early endosomes, the TGF-b receptor can be sorted to recycling endosomes and return back to the cell surface or it can be driven to late endosomes and lysosomes for degradation. The activated receptor complex recruits and phosphorylates the receptor-regulated SMAD (R-SMADs, e.g. SMAD2 and SMAD3). Association of TGF-b receptors with early endosomes adaptor proteins such as SMAD anchor for receptor activation (SARA), leads to the enhancement of TGF-ß -induced SMAD activation and the subsequent propagation of SMAD-dependent signaling. Activated R-SMADs dissociate from the TGF-β receptors and associate with Smad4, a common-partner for all R-Smads (Co-SMAD). These complexes translocate into the nucleus where they regulate transcription of TGF-b family target genes by binding transcriptional factors, transcriptional coactivators and corepressor. Posttranslational modifications of SMAD proteins, among which ubiquitination by NEDD4 family of E3-ligases, provide an additional level of regulation of the TGF-β signaling, controlling protein stability and function.

RNF11 emerges as a positive regulator of TGF-β signaling (Figure 2).

Several studies have reported that the overexpression of RNF11 enhances TGF-β signaling that is initiated at the receptor level and promotes TGF-β dependent migration in metastatic breast cancer. On the contrary, RNF11 silencing severely represses TGF-β responsiveness, downregulates the gene expression of several TGF-β responsive genes, and abolishes TGF-β dependent migration in metastatic breast cancer [13,15,29,38,40,65].

Data accumulated so far suggest that RNF11 could be involved at different stages of TGF-β signaling, concerning both the recruitment of trafficking machinery at the early endosomal compartment and regulation of HECT-E3 ligases activity.

It has been shown that RNF11 directly binds to the common (Co-)SMAD SMAD4, a protein that functions as a central transducer in the TGF-β responses. Following TGF-β receptors activation, SMAD4 forms heteromeric complexes with receptor activated regulatory (R-)SMADs. These complexes translocate into the nucleus, where they cooperate with other transcription elements to regulate the expression of target genes. Binding to RNF11 results in the stabilization of SMAD4 protein levels and the increase of SMAD4-dependent TGF-β signaling [65]. This effect could be achieved by two different mechanisms. It has been shown that the mono-ubiquitination of SMAD4 enhances its ability to oligomerize with R-SMADs promoting the subsequent TGF-β-induced transcription. On the contrary, polyubiquitination promotes SMAD4 degradation and the consequent termination of TGF-β signaling. Members of the NEDD4 family of E3-ligases, in particular SMURF1, SMURF2, NEDD4L, and WWP1, have been demonstrated to induce polyubiquitination-mediated degradation of multiple components of the TGF-β signaling pathway, including SMAD4 [66,67]. RNF11 might lower SMAD4 ubiquitination by inhibiting the activity of these E3-ligases that are involved in SMAD4 polyubiquitination. On the other hand, RNF11 interacts with SARA [57], an early endosome adaptor protein that promotes the activation of R-SMADs and, thus, boosts their binding to SMAD4. RNF11 would therefore promote the propagation of SMAD-dependent TGF-β signaling by lowering SMAD4 ubiquitination and by facilitating its association with SARA and the activated R-SMADs at the early endosomes [19,65].

It has also been reported that RNF11 binds to AMSH, a deubiquitinating enzyme that antagonizes the inhibitory effect exerted on the signaling pathway of TGF-β by the inhibitory (I-) SMADs, SMAD6 and SMAD7 [15]. The protein levels of AMSH decrease in presence of both RNF11 and SMURF2, and AMSH is ubiquitinated by SMURF2 in the presence of RNF11. This suggests that RNF11 recruits AMSH to SMURF2, leading to the ubiquitination and degradation of this DUB [35,62]. This mechanism could be particularly relevant in breast cancers, where it might counteract the positive effects of AMSH on TGF-β signaling and thus leading to cell proliferation and malignant progression. Indeed, AMSH mRNA has been found differentially expressed in breast cancer cell lines and the RNF11 protein has been found overexpressed in invasive breast cancers [33]. Finally, it has been shown that RNF11 outcompetes the I-SMAD SMAD7 in the binding to the HECT-E3 ligase SMURF2, blocking the SMURF2-mediated repression of TGF-β signaling by sequestering SMURF2 in an endosomal compartment [39].

In addition to regulating the degradation of R-SMADs, SMURF2 suppresses TGF-β signaling inducing the ubiquitin-dependent degradation of activated TGF-β receptors. As far as the mechanism is concerned, upon TGF-β stimulation, SMURF2 and SMAD7 form a complex in the nucleus, translocates in the cytosol, and is then recruited in the plasma membrane to activated TGF-β receptors. The SMURF2-SMAD7 complex generates an active E3-ligase that ubiquitinates activated TGF-β receptors, targeting them for degradation, and thus inducing TGF-β signaling termination. The SMURF2-RNF11 complex is a functional E3-ligase in vitro, whose activity is indistinguishable from the one of the SMURF2-SMAD7 complex, for both the E2 involved and the type of ubiquitin chain linkages generated. However, in vivo SMURF2 ubiquitination is completely abolished by RNF11 overexpression, resulting in the increase of TGF-b signaling [39]. This effect takes place through a mechanism that requires (i) the formation of the RNF11-SMURF2 complex, (ii) co-localization of RNF11 and SMURF2 to the endosomal compartment, and (iii) integrity and/or functionality of the RNF11 RING domain. The necessity of these conditions is supported by the evidence that the PPXY motif of RNF11 is necessary to assemble a stable complex with the WW3 domain of SMURF2 and the RNF11-Y40A mutant fails to reduce total SMURF2 ubiquitination, as well as to antagonize SMURF2-mediated repression of TGF-β responsiveness. A similar effect can be observed with the RNF11 mutant that is inactivated in the palmitoylation site that is sequestered in the Golgi and, thus, impaired in SMURF2 binding. The RNF11 mutants inactivated in the sorting signals (i.e. myristoylation, ubiquitination, and di-leucine motifs) bind to SMURF2, but are impaired in the ability to extinguish SMURF2 ubiquitination [38]. This suggests that the localization of RNF11, which cycles between the plasma membrane and the endosome compartment after ligand stimulation, could be a critical element that allows for RNF11 to switch between the functions of inhibitor and enhancer of SMURF2 ubiquitination, acting respectively as a positive or negative regulator of TGF-β signaling.

Finally, both RING domain and E2 binding mutants of RNF11 interact with SMURF2 and downregulate its ubiquitination. However, they are not able to suppress the SMURF2-mediated repression of TGF-β responsiveness, suggesting that integrity and/or function of RNF11 RING domain are somehow involved in the mechanism through which RNF11 antagonizes the function of SMURF2 [38].

In analogy with what has been observed for SMURF2, RNF11 binds and inhibits other NEDD4 E3 ligase family members that are implicated in the TGF-β pathway [40], further underlining how RNF11 enriches the complexity of the ubiquitin ligase network that interferes with this signaling pathway.

#### 2.4.2. The EGF Receptor Signaling Pathway

**Figure 3:**
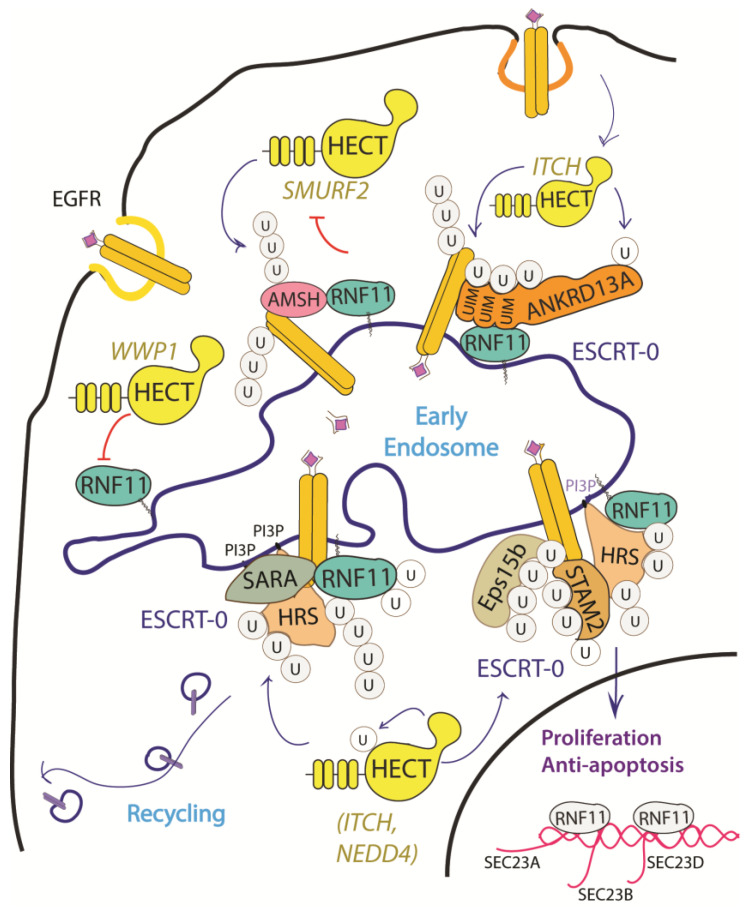
Graphical representation of the role of RNF11 in the EGFR signaling pathways. The Epidermal Growth Factor (EGF) receptor is a membrane tyrosine kinase that initiates cell growth and proliferation. Following ligand binding, the EGF receptor undergoes endocytosis via clathrin-dependent and clathrin-independent pathways. Following ligand stimulation, the EGF receptor undergoes endocytosis via clathrin-dependent and clathrin-independent pathways. Both routes require essential ESCRT-0 components, such as EPS15 and Epsin, which recognize the ubiquitin moieties conjugated on the receptor cytoplasmic tails following ligand stimulation. The ANKRD13 family of proteins could act as alternative ESCRT components that drive the receptor towards the endocytic routes.

The EGF receptor is a membrane tyrosine kinase that initiates cell growth and proliferation. Once activated by ligand binding, EGFR is rapidly internalized and routed to lysosomes for degradation. As previously described, receptor ubiquitination is a crucial signal that commits EGFR to lysosomal degradation and allows for signal termination. The deregulation of EGFR signaling is indeed a significant feature in different stages of oncogenesis and in several types of cancer [68]. Evidence accumulated so far implicated RNF11 in the downregulation of EGFR signaling by promoting lysosomal degradation (Figure 3).

The depletion and overexpression of RNF11 have both been shown to impair ligand-induced EGFR degradation and to increase EGFR recycling [32]. This suggests that RNF11 exerts its function in the early phases of receptor endocytosis, during which endosomal sorting occurs and EGFR is committed for degradation or recycling. Moreover, RNF11 overexpression causes a reduction of EGFR levels and receptor hyper-ubiquitination following EGF induction [31].

Several mechanisms have been investigated in order to clarify the role of RNF11 in the EGFR pathways, however a comprehensive framework that integrates all of them is still missing (Figure 3). RNF11 binds the DUB protein AMSH and promotes its degradation by the E3-ligase SMURF2-mediated ubiquitination [15]. It has been shown that AMSH interacts with the ESCRT-0 STAM-1 subunit and favours recycling of activated EGFR by promoting receptor deubiquitination. Coherently, AMSH knockdown enhances EGFR degradation [69]. Therefore, RNF11-mediated AMSH degradation might increase EGFR ubiquitination, clearly committing activated receptor to lysosomes.

Different HECT ligases have been demonstrated to inhibit EGFR degradation and promote cell proliferation. In particular, WWP1 upregulates EGFR levels in a ligase activity independent manner [39]. RNF11 associates with WWP1 and the two ligases reciprocally ubiquitinate each other in cells without affecting their degradation. The transient overexpression of WWP1 promotes cell proliferation, while WWP1 knockdown significantly suppresses cancer cell proliferation and induces apoptosis [40,61]. Interestingly, the depletion of RNF11 neutralizes the decrease of EGFR levels that are induced by WWP1 knockdown, which suggests that WWP1 could promote cell proliferation by inhibiting RNF11 [39,70]. Conversely, NEDD4 and ITCH HECT-E3-ligases have been demonstrated to inhibit EGFR degradation by ubiquitinating multiple adaptor proteins essential for the endocytosis of ligand-activated EGFR [18,62,71,72,73,74]. Again, RNF11 might counteract this by inhibiting HECT-E3 ligases activity.

We have investigated a similar model on the adaptor protein ANKRD13A. Soon after EGF stimulation, ANKRD13A binds ubiquitinated EGFR and acts as molecular scaffold that promotes, within 5–10 min. from induction, the transient formation of a complex between activated EGFR and the E3-ligases ITCH and RNF11. Both of the ligases contact the UIMs region of ANKRD13A. Nevertheless, while ITCH promotes the ubiquitination of ANKRD13, RNF11 acts rather as a negative regulator of this modification. As previously discussed, the ubiquitination status of ANKRD13A strictly regulates its ability to bind activated EGFR and thus to mediate receptor rapid internalization.

Finally, RNF11 seems to be involved in the transport of newly synthetized EGFR to the cell surface that is induced by prolonged EGF ligand stimulation and it is aimed at restoring EGFR plasma membrane levels [34]. EGFR prolonged activation increases the expression of essential components of coat protein complex II (COPII)-coated vesicles—specifically SEC23A, SEC24B, and SEC24D—that take part in the ER-to-Golgi trafficking of the newly synthesized receptor molecules along the secretory pathway. RNF11 overexpression has been shown to specifically increase the mRNA levels of SEC23B, SEC24B and SEC24D. Coherently, RNF11 knockdown reduces the mRNA levels of these proteins and the number of ER exit sites, leading to a significant decrease of the EGFR transport efficiency. Notably, after prolonged EGF stimulation, RNF11 translocates from the cytoplasm to the nucleus, which suggests a possible direct role in the transcription of these genes.

#### 2.4.3. The NF-kB Signaling Pathway

A third pathway that involves RNF11 is the Nuclear factor-κB (NF-κB)/Rel signaling, which controls genes involved in a broad range of biological processes, from immunity and inflammation to cell survival, in response to a wide range of stimuli [75]. In this context, RNF11 is involved as a negative regulator in the termination of the inflammatory response functioning.

**Figure 4:**
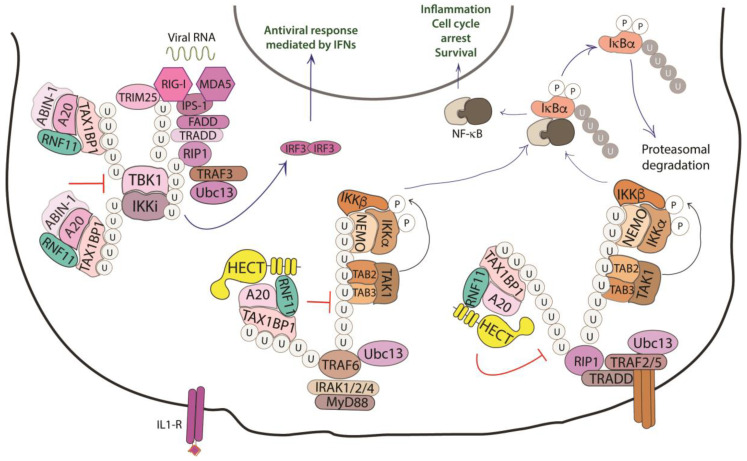
Role of RNF11 in the NF-κB signaling pathway. The activation of the NF-κB signaling leads to the degradation of the inhibitory IκB proteins (IκB, inhibitor of NF-κB), that mask the nuclear localization signals required for the nuclear import of NF-kB. The downstream recruitment of most of the signaling proteins is mediated by poly-ubiquitination of several components of the signal transduction machinery. E3-ligases, such as TRAF2/3/5/6 ubiquitinate the receptor interacting protein 1 (RIP1) by K11 and K63-linked ubiquitin chains. These chains serve as a scaffold for the recruitment of the linear ubiquitin chain assembly complex (LUBAC) that mediates NEMO M1-linear ubiquitination. In turn, it allows for the recruitment of TGF-β Activated Kinase 1 Binding Protein 2/3 (TAB2/3) and TAK1 that activate the IKK complex (IKK1/IKK2/NEMO). This complex triggers the ubiquitin-dependent degradation of IκB and the release of the NF-κB transcription factor. The deubiquitinating enzymes counteract the activity of the E3-ligases and play an essential function in restricting NF-kB signaling by targeting critical pathway components. In particular, the A20 ubiquitin-editing complex, comprising A20, TAX1BP1, ABIN-1, ITCH, and RNF11, catalyzes the removal of K63-linked ubiquitin chains from proteins of the signal transduction machinery, and the subsequent addition of K48-linked ubiquitin chains, promoting degradation by proteasome and termination of NF-κB signaling.

NF-κB proteins function as dimeric transcription factors that are maintained inactive in the cytoplasm by their interaction with inhibitory IκB proteins (IκB, inhibitor of NF-κB), which mask the nuclear localization signals that are required for the nuclear import of NF-κB dimers. Signaling through a subset of receptors induces the release of the inhibitory mechanism and creates transcriptionally competent NF-κB complexes that translocate to the nucleus and induce the expression of target genes (Figure 4).

Whilst ligand-induced phosphorylation events promote binding to the activated receptors, the poly-ubiquitination of several components of the signal transduction machinery acts as a binding platform that mediates the downstream recruitment of most of the signaling proteins [76]. In particular, the protein family of tumor necrosis factor (TNF) receptor-associated factor (TRAF) E3-ligases mediates K63-linked ubiquitination of many of these signaling proteins. Deubiquitinating enzymes counteract the activity of the E3-ligases and play an essential function in restricting NF-kB signaling by targeting critical pathway components [77].

A20 is one of these DUBs, a deubiquitinating enzyme that is transcriptionally upregulated by NF-κB, which works as a ubiquitin-editing enzyme. Indeed, A20 removes Lys63-linked chains from substrates and promotes the conjugation of Lys48-linked chains that lead to proteasomal degradation of targets [78]. The activity of A20 is carried out together with the adaptor Tax1 Binding Protein 1 (TAX1BP1) and the E3-ligase ITCH [79]. TAX1BP1 functions as an adaptor that recruits ITCH to A20 and A20 to its substrates, which are the receptor interacting protein 1 (RIP1) and the E3-ligase TRAF6. Even though the precise role of ITCH in the A20 complex is still unclear, it is possible that this HECT-ligase collaborates in the ubiquitin editing function of A20 by ubiquitinating RIP1 with K48-linked ubiquitin chains [80]. Harhaj and colleagues have shown that RNF11 transiently associates with A20, TAX1BP1, and RIP1 following TNF stimulation and that RNF11 knockdown abrogates the assembly of the A20 complex on RIP1, which causes persistent phosphorylation of the inhibitor protein IkBα and c-Jun N-terminal kinase (JNK) [79,81]. Moreover, when RNF11 is knocked down, RIP1 and TRAF6 ubiquitination are significantly enhanced upon ligand stimulation, which indicates that RNF11 antagonizes the stimulus-dependent ubiquitination of both proteins.

The knockdown of endogenous RNF11, as well as the expression of ectopic RNF11, also inhibits IL-1 and poly (I:C)-induced NF-kB activation, which indicates that RNF11 is essential for the inactivation of key signaling molecules in both the TNFR and TLR4/IL-1R pathways. Later, publications have confirmed that the reduced expression of RNF11 results in the aberrant regulation of inflammatory signaling in neuroblastoma cells, microglia cell line, and murine cortical neurons, which suggests a tight association of RNF11 with the pathogenesis of neurodegenerative diseases [79,82,83,84].

RNF11 function has also been investigated in the innate antiviral signaling pathways that lead to the production of type I interferons (IFNs) [85]. This pathway implicates the TRAF3-mediated Lys63-linked polyubiquitination of non-canonical IκB kinase IKKi and TANK-binding kinase 1 (TBK1). IKKi and TBK1 ubiquitination promotes the activation of kinases, which, in turn, phosphorylate the transcription factor IRF3, triggering its dimerization and nuclear localization, followed by the induction of type I IFN. It has been demonstrated that the overexpression of RNF11 abrogates the interaction between TRAF3 and TBK1/IKKi and disrupts the Lys63-linked polyubiquitination of TBK1 and IKKi, thus counteracting IFN-β activation.

Not surprisingly, RNF11 has been reported to be a target of different miRNAs previously demonstrated to enhance or repress expression of pro-inflammatory mediators of NF-κB signaling. Among them, miR-146a induction by NF-κB represses Toll-like receptor signaling acting on TRAF6, IRAK1/2, and RNF11 [86,87,88]. Conversely, the positive regulation of NF-κB signaling by miR-19b involves the coordinated suppression of A20 and RNF11 [89,90]. All of this being, data support the notion that RNF11 acts at an early stage of the inflammatory response by interfering with the ubiquitination events leading to the recruitment of adaptors and transducers of the signaling pathway. It is conceivable that the release of the inhibitory function of RNF11, which is driven by unidentified factors, could play a role in determining the timing of the signal transduction process.

### 2.5. RNF11, a Novel Regulator of E2s and E3s Activity

Numerous regulative mechanisms, extensively reviewed in the recent years [20,55,91], control the catalytic activity of the E3-ligases in terms of interaction with the E2, recognition of the substrate, and assembly of a specific chain. These mechanisms include post-translational modifications—mainly phosphorylations and conjugation with ubiquitin and ubiquitin-like proteins—and the noncovalent binding of proteins. The common element is represented by the fact that each enzyme is kept in a closed and inactive conformation, until a given signal triggers the release of the inhibitory state and the consequent access to the protein substrates [92,93,94,95]. Although still far from being conclusive, the experimental data accumulated so far suggest three principal roles for RNF11: as HECT substrate, HECT adaptor, and HECT inhibitor. As previously discussed, RNF11 is a substrate of HECT ligases for poly-ubiquitination reactions. The interaction with members of the NEDD4 family has been extensively analysed by several groups and the binding sites have been mapped, which confirms the WW/PPxY recognition as the main interaction mode. Accordingly, the mutation of the PY motif in RNF11 disrupted the interactions with the NEDD4 family members and stabilized RNF11 mutant protein levels, clearly indicating that at least some NEDD4 family members control RNF11 stability [17]. Notably, the ubiquitination of the RNF11 PY mutant is not completely abrogated and still depends on the catalytic activity of the NEDD4 family proteins. The presence of secondary contact sites [17] might be important in differentiating between degradative or regulatory functions of RNF11 ubiquitination.

Data reporting the involvement of RNF11 in HECT-mediated ubiquitination pathways share the common evidence of RNF11 acting as an adaptor of HECT ligases, although this function seems to be broader and more complex than is currently known. In fact, adaptor proteins of HECT ligases typically serve the double function of acting as a bridge for the substrate recruitment and as a regulator of the catalytic activity. The adaptor function of RNF11 has been clearly demonstrated for GGA3 protein, which is ubiquitinated by ITCH in an RNF11-dependent manner and, in principle, this mechanism can be extended to all of the GGA family members, because the VHS domains embedded in these proteins are able to bind the RNF11 di-leucine motifs. The ability of RNF11 to interact with several ubiquitin binding motifs would further extend its adaptor function, thus widening the spectrum of substrates that are recruited by the HECT ligases. Moreover, the kinetic of association between RNF11 and many of its validated binding partners shows to be sensitive to the stimulation of several membrane receptors, with a transient increase in binding ability detectable at the early phases of induction. For example, binding of RNF11 to TRAF6 occurs 15–30 minutes after IL-1 stimulation, and then RNF11 is released [96]. A similar kinetic can be observed in the interaction with the E2 enzymes UBE2N and UbcH5, which cooperate with TRAFs and cIAPs in the TNFR signaling pathway [97,98]. Similarly, the transient complex, including RNF11, ANKRD13A, and the activated EGFR, is assembled and persists until the receptor reaches the early endosome compartment, within 15–20 minutes from plasma membrane invagination, after which RNF11 is released [31]. These observations suggest that the RNF11 adaptor function is strictly regulated in space and time in order to limit the interaction with substrates and/or chain elongation on the target.

RNF11 emerges as a regulator of the NEDD4-like HECT ligases. In vivo, RNF11 overexpression strongly inhibits the ITCH-mediated ubiquitination of several protein adaptors (GGA, HRS, STAM2, ANKRD13A) [18,31], as well as the ubiquitination of SMURF2 or WWP1 [38,39]. In some cases, this ability is strictly dependent on the functionality of the RING-H2 domain, while, in other cases, it is not. Moreover, there is evidence that in vitro RNF11 assembles an active E3-ligase complex with SMURF2, while the in vivo function of this complex is strongly dependent on the sorting determinants that govern the subcellular localization of the RING ligase [38]. This variability suggests the existence of different RNF11-mediated inhibitory mechanisms [38].

Whatever the mechanism, this function must require (i) the recruitment of the HECT ligase in an open conformation by disrupting the intra-molecular inhibition, (ii) the binding of a subset of substrates, and (iii) a molecular switch that allows for substrate ubiquitination by the HECT ligase only after an unidentified signal triggering the dissociation of RNF11 arrives.

**Figure 5:**
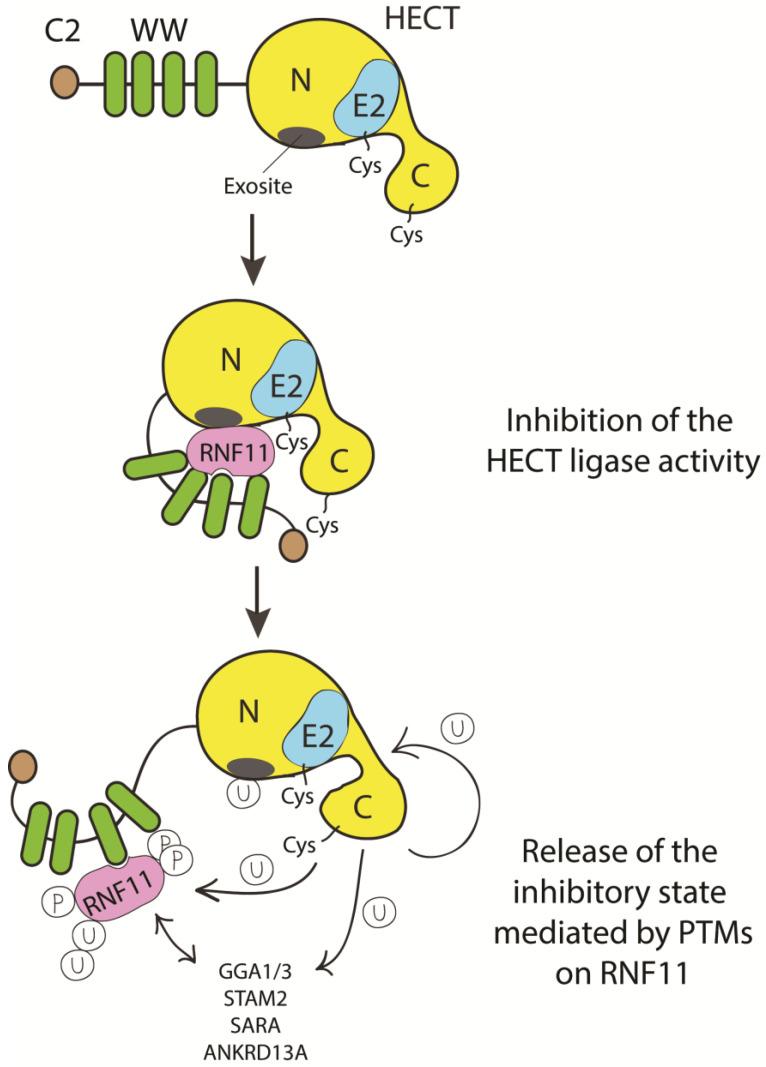
Cartoon representing putative inhibitory mechanisms of HECT ligases mediated by RNF11. E3-ligases of the HECT family are characterized by the presence of C2 and WW domains located upstream of the catalytic domain. RNF11 binds the WW domain containing region of all the HECT family members and these interactions are reported to inhibit HECT activity. This inhibition can result from several events. Here, we suggest that RNF11 promotes a closed conformation, which masks the E2-binding site and/or the non-covalent ubiquitin binding site (exosite) on the HECT ligase. Post-translational modifications modulated by the activation of different signaling pathways could be responsible for the conformational modifications of RNF11, allowing the recruitment of substrates on the WW domains and triggering the adaptor functions of RNF11.

To date, we can only speculate on how HECT inhibition by RNF11 can be achieved (Figure 5).

We know that RNF11 binds the WW3 domain of ITCH, NEDD4, and SMURF2 [17]. Several HECT E3s are kept in a catalytically inactive state by intramolecular interactions between the N-terminal region (either the C2 or the linker region between the WW domains) and the C-terminal HECT domain. By interacting with the WW3 domain, RNF11 could therefore modulate the inhibitory mechanism of selected NEDD4 family members. RNF11 could also prevent the interaction of the HECT ligase with the E2 enzyme. It has been shown that RNF11 binds the E2 UBC13 so tightly in order to outcompete both the E1 enzyme and an active E3 ligase. In addition, RNF11 binds the UBC13-Ub intermediate tightly, but it does not promote the assembly of ubiquitin chains [12]. Thus, by limiting the availability of the E2 enzyme, RNF11 may regulate the activity of E3 that relies on that E2. Notably, since UBC13 is the only E2 enzyme known to be capable of catalyzing Lys63-linked polyubiquitination, RNF11 could regulate the activity of E3s that rely on UBC13 (es. TRAF2/6, RNF8), acting rather like an inhibitor of the assembly of K63-linked ubiquitin chains [12]. This function might not be limited to UBC13 partners. ITCH, whose activity is inhibited by RNF11 [17,18], mainly assembles K63-linked chains and forms K48 chains only to a much lesser extent in vitro [94,99]. However, at least in some cases, ITCH-mediated proteasomal degradation of specific targets (i.e. TXNIP protein) requires the formation of K48/K63 branched chains [99]. Therefore, it would be possible that, by inhibiting Lys63-chain elongation, RNF11 indirectly favors the assembly of K48-linked ubiquitin chains, and eventually branched ubiquitin chains. This might be a more general function of RNF11, given that other members of the NEDD4 family also preferentially form K63-linked chains, but can also assemble K48-linked chains [100].

Alternatively, or in addition, considered its ability to be directly bound by ubiquitin binding domains, RNF11 could mask the exosite, a non-covalent ubiquitin binding site in the N-lobe of the HECT domain that appears to be required for enzyme processivity [93,101,102,103]. In this way, RNF11 might prevent HECT poly-ubiquitination activity by contacting the exosite in a manner resembling the binding of ubiquitin. The binding to the exosite would prevent the HECT ligase processivity, thus acting through the same molecular mechanism in different signaling pathways. The experimental evidence indicating RNF11 as a potential “mimic” of ubiquitin support this intriguing hypothesis [31].

Additionally, RNF11 could recruit the E2 enzyme that cooperates with the HECT ligase, though maintaining it in an inactive conformation until a specific signal promotes the release of the inhibitory mechanism. We can suggest that the transition requires a change in the post-translational modification profile of RNF11, which is by a specific signal leading to RNF11 dissociation, chain extension and substrate degradation. This rearrangement could switch the function of RNF11from being a HECT-E3-ligase inhibitor to acting as an E4 enzyme promoting the polyubiquitination and degradation of substrates.

Finally, RNF11 has been also shown to interact with Cullin1, a core member of the SCF (Skp1/Cul1/F-box protein) complex. This suggests that RNF11 could potentially also act as an adaptor and/or inhibitor of Cullin RING ligase complexes in analogy with the mechanism that is described for HECT ligases [40].

In this framework, RNF11 appears to be a multifaceted RING-H2 protein having predominantly E3-independent functions, but whose activity is intimately linked to a wide array of different E2 and E3 enzymes. We expect that further studies will prove to be useful in disclosing key elements that are essential to improve our understanding of this molecular mechanism.

### 2.6. Pathogenic Significance of RNF11 and Future Perspectives in Target Therapy

The work carried out by several groups in recent years has gradually revealed the ability of RNF11 in order to modulate in space and time the ubiquitination events of crucial cellular pathways, specifically TGF-β, EGF, and NF-κB signaling. This may be particularly relevant for pathological situations.

To date, the only report of a phenotypic effect associated with RNF11 mutations concerns a c124-2A > G splice variant identified in Belgian Blue Cattle (BCC). This is a loss of function mutation that is responsible for stunted growth and disease resistance. Approximately one third of homozygous mutant calves die prematurely before six months of age with major inflammatory lesions. However, frequency of carriers in the BCC population is surprisingly high, which suggests the possibility that a phenotypic benefit caused a selective sweep [104].

The involvement in the A20 ubiquitin-editing complex, which turns off the signaling cascade, leading to NF-κB activation [13], also suggests a role for RNF11 in the dysfunction of neuronal cells [83,105]. Persistent NF-κB activation is a hallmark of neurodegenerative diseases, and the dysfunction of the ubiquitin-proteasome system in the substantia nigra of Parkinson disease (PD) patients is part of the pathogenic process [106]. RNF11 gene expression and the protein level are decreased in the substantia nigra of brain tissues affected by PD [15,84,107]. In addition, RNF11 turned out to be sequestered into Lewy bodies, the histopathological landmarks of PD, and in patients with Alzheimer disease with Lewy pathology, where it colocalizes with α-synuclein [108,109].

The role of RNF11 in counteracting the persistent NF-κB activation has been validated in several cell lines, particularly in neuroblastoma cells, primary cortical neurons and microglial cells, where it has been shown to protect against LPS-induced cell cytotoxicity [82]. Moreover, RNF11 gene results in being upregulated, in combination with A20, in the hypothalamus of mice treated with high fat diets. This possibly provides protection for neurons from non-regulated inflammation as it immediately counteracts the activation of NF-κB signaling induced even after three days of high fat diet. On the other hand, RNF11 interacts with melanocortin 3 and 4 receptor (MC3/4R) and its overexpression results in the reduction of the signaling of these important regulators of body weight and energy consumption [110]. A decrease in the function of MC3R and MC4R strongly contributes to the development of leptin resistance and, later on, to the pathogenesis of obesity [110]. Thus, RNF11 might represent a critical element in the neuronal pathways that are involved in weight regulation, linking melanocortin signaling and hypothalamic inflammation.

High levels of RNF11 have been reported in breast, prostate, colon, and pancreatic cancers [33,111]. During the early stages of tumorigenesis, RNF11 overexpression appears to act as an inhibitor of cellular proliferation and, thus, counteracts tumor growth [13,31,33,38,39,65]. However, at later stages of tumorigenesis, RNF11 seems to promote the migratory and invasive properties of cancer cells by inducing the epithelial-mesenchymal transition (EMT) [112]. RNF11 disruption by insertional mutagenesis and knockdown both impair the metastatic potential of murine melanoma B16F10 cells in vitro and in vivo, respectively. Consistently, RNF11 overexpression increases the migratory potential of melanoma cells [113]. Moreover, RNF11 knockdown reduces cell migration and proliferation in the 4T1 and 4T07 breast cancer cell lines, and specifically decreases the expression of genes promoting tumor growth and metastasis [38].

All of this being considered, these data strongly suggest that targeting RNF11 may be therapeutically beneficial, especially in multiple types of cancer. After all, E3-ubiquitin ligases are promising candidates for pharmacological cancer therapies and small molecule inhibitors of some of them have already been developed [114,115,116]. The recent report that, in two different gastric cancer cell lines, SNU5 and NCI-N87, the knockdown of RNF11 increases the sensitivity to gefitinib treatment strongly suggests RNF11 as a promising therapeutically exploitable target [117]. Moreover, the evidence that RNF11 silencing only confers a significant therapeutic benefit in gastric cell lines with high RNF11 expression (SNU5 and NCI-N87), but not in those with low RNF11 expression (SNU638), suggests that the transcriptome expression of RNF11 might be a potential predictor of drug sensitivity [117].

## Figures and Tables

**Table 1:** Post-translational modifications experimentally validated or identified by prediction servers in human and mouse RNF11 homologues.

PTM	Residue	In Vivo Identification	Reference (PMID)	Predicted	
				NetPhos3.1	UbPred
Myristoylation	Gly2	✓	20676133		
Palmitoylation	Cys4	✓	20676133		
Nitrosylation	Cys4	✓	24105792		
Ubiquitination	Lys6				✓
	Lys82	✓	22790023, 32274752		
Sumoylation	ND	✓	32274752		
Phosphorylation	Ser7			✓	
	Ser10	✓	22617229	✓	
	Ser14	✓	27281782, 27251275,	✓	
			25850435, 24275569,		
			23749302, 23312004,		
			23186163		
	Ser19			✓	
	Ser21	✓	20415495	✓	
	Ser25	✓	25850435, 23684622, 19144319	✓	
	Thr30	✓	24275569	✓	
	Thr52			✓	
	Ser54	✓	24275569	✓	
	Thr63	✓	22645316	✓	
	Ser92			✓	
	Thr135	✓	16123141		
	Ser149			✓	

Experimentally validated data were extracted from PhosphoSite-Plus (https://www.phosphosite.org/homeAction.action) and Phospho-ELM (http://phospho.elm.eu.org) databases, while NetPhos3.1 (http://www.cbs.dtu.dk/services/NetPhos/) and UbPred (http://www.ubpred.org) predictors were used to identify putative modification sites. For details refer to the text. ✓ indicates residues that have been found phosphorylated in vivo or predicted to be post-translationally modified by predictor software. ND = not determined.

**Table 2:** List of RNF11 binding partners identified by Y2H-screeening (violet) and mass spectrometry (orange) experiments. When known, the domain selected in the Y2H screening is indicated. Proteins have been clustered according to their main biological role. Proteins that have been identified in both systems are shown in bold.

Yeast-Two-Hybrid			Mass Spectr.	Yeast-Two-Hybrid		Mass Spectr.
Gene Name	Binding Domain	Gene Name	Gene Name	Gene Name	Binding Domain	Gene Name
(A) E2, E3 and DUBs				(C) Effectors of anti-inflammatory response		
**UBE2N**	UBC	UBCH5c		TNFAIP3		IKBKB
UBCH5a	UBC	UBCH6		TAX1BP1	CC-ZnF_C2H2	CHUK
UBCH5b	UBC			OPTN	ZnF_C2H2	S100A8
UBCH5c	UBC			NEMO	UBAN-ZnF_C2H2	S100A9
HERC1	UBA-like			RNF216	IBR	S100A11
HERC2	UBA-like	UBCH7		(D) Membrane proteins and receptors		
HUWE1		UBCH9				
CBL-b	UBA	UBC7		CD45		EGFR
**WWP1**	WW	UBE2V1				TGFBR1
**WWP2**	WW	hRul138				TFRC
**NEDD4**	WW	LISTERIN				CD44
**NEDD4-2**	WW	**ITCH**				ATP2B1
**SMURF1**	WW-HECT					IFITM3
**SMURF2**	WW-HECT					RTN4
USP5	UBA			(E) Other functions		
TNFAIP3	ZnF_A20					
RNF168	UIM-MIU			NY-REN-25	UBD	MYOF
USP5				ERCC6	UBD	MYOF
AMSH				QARS		DAP3
CUL1				POLI		PDXP
(B) Effectors and adaptors of intracellular traffic				KHNYN	NYN-CUBAN	SLC27A2
				CALCOCO2	ZnF_C2H2	PTP4A1
RPS27A		RAB6A		AUP1	CUE	PHB
UBA52		EXOSC10		SPRTN	UBZ	PHB2
EPN1	UIM			RP42	UBA	APOA2
EPN2	UIM			FLJ21588	CUE	SHROOM3
EPN3	UIM			SDCBP	PDZ	CHCHD3
ANKRD13A	UIM			SMAD4		QPCTL
GGA1	GAT					TXN
GGA3	GAT					LRRC59
TOM1L2	GAT					SOAT1
ALIX						MOGS
EPS15						BRI3BP
STAM2	VHS-UIM-SH3-GAT					DRG1
Endofin						VCP
ALIX	ALIX-LYPXL-bnd					DMD
SARA						MSN
UBQL-2	UBA					
UBQL-4	UBA					
MYO6	MIU-MyUb					
NAF1						
RABEX5						
NDP52						
